# Exercise Activates p53 and Negatively Regulates IGF-1 Pathway in Epidermis within a Skin Cancer Model

**DOI:** 10.1371/journal.pone.0160939

**Published:** 2016-08-10

**Authors:** Miao Yu, Brenee King, Emily Ewert, Xiaoyu Su, Nur Mardiyati, Zhihui Zhao, Weiqun Wang

**Affiliations:** 1 Department of Food Nutrition Dietetics & Health, Kansas State University, Manhattan, Kansas, United States of America; 2 Institute for Agri-food Standards and Testing Technology, Shanghai Academy of Agricultural Sciences, Shanghai, China; Universidad Pablo de Olavide, SPAIN

## Abstract

Exercise has been previously reported to lower cancer risk through reducing circulating IGF-1 and IGF-1-dependent signaling in a mouse skin cancer model. This study aims to investigate the underlying mechanisms by which exercise may down-regulate the IGF-1 pathway via p53 and p53-related regulators in the skin epidermis. Female SENCAR mice were pair-fed an AIN-93 diet with or without 10-week treadmill exercise at 20 m/min, 60 min/day and 5 days/week. Animals were topically treated with TPA 2 hours before sacrifice and the target proteins in the epidermis were analyzed by both immunohistochemistry and Western blot. Under TPA or vehicle treatment, MDM2 expression was significantly reduced in exercised mice when compared with sedentary control. Meanwhile, p53 was significantly elevated. In addition, p53-transcriptioned proteins, i.e., p21, IGFBP-3, and PTEN, increased in response to exercise. There was a synergy effect between exercise and TPA on the decreased MDM2 and increased p53, but not p53-transcripted proteins. Taken together, exercise appeared to activate p53, resulting in enhanced expression of p21, IGFBP-3, and PTEN that might induce a negative regulation of IGF-1 pathway and thus contribute to the observed cancer prevention by exercise in this skin cancer model.

## Introduction

Physical inactivity, together with overwhelming calorie intake, is a main etiological factor that contributes to cancer development [1]. Scientific evidence for primary cancer prevention through physical activity is accumulating promptly. To date, large epidemiological studies and clinical trials have demonstrated that physical activity is effective in reducing the risk of various cancers, including breast, colon and colorectal, pancreatic, prostate, endometrial, ovarian, and lung cancer, with a reduction rate of 10–50% [2–4]. A cohort study with 45,631 U.S. women and an average follow-up of 8.9 years, for example, observed statistically significant decrease in breast cancer risk among those who exercised moderately (>10 hours per week of hiking or walking), when compared to no hiking or walking group [5]. In addition to human studies, exercise intervention trials have been conducted in animal models to show a protective role against PTEN-deficient mouse liver tumors [6], DMH-induced rat colon cancer [7], and UVB-induced mouse skin carcinogenesis [8]. Our previous studies conducted in a mouse skin cancer model showed that treadmill exercise with iso-caloric intake of the sedentary counterpart could prevent cancer risk significantly [9–10].

The mechanism by which exercise may reduce cancer is not well defined. Previous studies by us and others have found that exercise-induced cancer prevention is associated with a reduction of multiple hormones, especially for the mitogenic hormone IGF-1 [9, 11]. IGF-1 has been related to enhancement of cell proliferation and angiogenesis, and inhibition of apoptosis [9]. During mitogenesis, IGF-1 exerts its function through cell signaling networks resulting in the activation of both IGF-1-dependent MAPK-proliferation and PI3K-anti-apoptosis pathways for cancer promotion [12–14]. Furthermore, IGF-1 signaling could be activated by 12-*O*-tetradecanoylphorbol-13-acetate (TPA). TPA, a phorbol ester, is commonly used to promote skin carcinogenesis in mouse model [15]. When applied to mice epidermis, TPA stimulates phospholipid dependent serine/threonine kinase such as protein kinase C (PKC) [16], and then activates the Ras-regulated downstream networks, particularly the IGF-1 dependent MAPK cascade, leading to tumor cell growth [17–18]. Our previous studies conducted in TPA-promoted mouse skin cancer model have demonstrated that the reduction of IGF-1 in response to exercise-induced weight loss corresponded to a concomitant inhibition of IGF-1-dependent mitogenic cascades and thus diminution of TPA-promoted signaling [9–14]. Considering the significant role of IGF-1 in cell growth and survival, inhibiting TPA-promoted IGF-1 signaling appears to be a critical target for cancer prevention. However, the possible up- and down-stream proteins that regulate exercise-induced IGF-1 pathway reduction have yet to be identified. Some studies have suggested a potential connection between p53 and IGF-1 pathways in dietary caloric restriction-induced life extension [19]. Leung et al. found exercise significantly decreased the IGF-1 axis in vivo and increased p53 protein in in vitro prostate tumor cells, suggesting a potential down-regulation of IGF-1 by p53 [20]. As a tumor suppressor, p53 is essential for gene stabilization and DNA repair. On the other hand, p53 is also a transcription factor that has a sequence-specific DNA-binding domain in the central region and a transcription activation domain at N terminus [21]. In normal cells, p53 is maintained at a low concentration by its negative regulator MDM2 protein, a ring finger ubiquitin ligase that can bind with p53, forming a complex that triggers p53 degradation [22–23]. However, upon DNA damage, p53 is released from MDM2 through phosphorylation [24]. Activated p53 then selectively stimulates the transcription of its target genes, including p21, IGFBP-3, and PTEN, to initiate cellular apoptosis and cell cycle arrest [25–27]. It has been documented that p53 is effective in protecting UVB-induced skin cancer in *p53* knockout mice [28]. The current study is aimed to investigate the cancer preventive mechanisms of moderate exercise by a potential down-regulation of IGF-1 signaling which activates p53 in TPA-promoted SENCAR mice. The precursor regulator of p53 and p53-transcriptioned proteins associated with IGF-1 pathway regulation were systematically examined.

## Materials and Methods

This study was approved by Kansas State University IACUC with protocol #3019.0

### Animals and treatment

Forty Six-week old female SENCAR mice from National Institutes of Health (Frederick, MD) were housed individually at 24 ± 1°C with a 12:12 light-dark cycle. Mice were randomly assigned into one of two groups: *ad libitum* feeding sedentary control and exercise but pair feeding of the same amount as sedentary control (AIN-93 diet). After a two-week training period for adaptation to the new environment or treadmill exercise accordingly, animals in exercise group were placed on a zero-grade adjustable-speed mouse 5-lane treadmill (Harvard Apparatus, Holliston, MA) at 20 m/min, 60 min/day and 5 days/week for 10 weeks. At the end of the experiment, the mice dorsal skin was shaved and applied with acetone only or 3.2 nmol TPA dissolved in 200 μL acetone. After two hours, the mice were treated with diethyl ether and sacrificed by decapitation. Skin tissues were then snap-frozen in liquid nitrogen and stored at -80°C for further analysis of targeted proteins by immunohistochemistry (IHC) and Western Blot.

### Protein analysis by IHC

The frozen dorsal skin tissues were fixed in 3.7% formalin for 22 hours at -70°C and then switched to 70% ethanol. The skin tissues were set on edge in paraffin blocks, sectioned at a thickness of 4 micrometers, and then placed on slides. For immunostaining, sections were deparaffinized twice in Master Clear (American MasterTech Scientific, Lodi, CA) for 10 min, followed by rinsing in absolute alcohol for 1 min. Slides were subsequently placed in 30% H_2_O_2_ in methanol for endogenous peroxidase quenching for 20 min. Sections then underwent rehydrated through 100%, 95%, 75%, and 50% ethanol, respectively, and were finally placed in distilled water. Antigens were retrieved by steaming in citrate buffer for 20 min. After PBST washes, sections were incubated overnight at 4°C with 1:160 dilution in PBST for p53 primary monoclonal antibody (Cell Signaling Technology, Danvers, MA) and 1: 50 dilution for other antibodies against mouse MDM2, IGFBP3, PTEN (Cell Signaling Technology, Danvers, MA), respectively. Secondary antibody Alexa Fluor 555 IgG (Life Technologies, Carlsbad, CA) were applied with a dilution of 1:500 at 37°C for 60 min. Images were obtained using a LSM-5 confocal microscopy and analyzed using Zeiss Image J software (Jena, Germany). Quantification of the IHC images were calculated by using sample’s image minus negative control’s to eliminate the background stain. A negative control was treated with secondary antibody but not primary antibody, while a sample was treated with both primary and secondary antibody.

### Protein analysis by Western Blot

Mouse skin tissues were sliced and placed in 600 μL ice-cold PBS containing PMSF (1:100). After sonicating for 15 s, samples were centrifuged at 7,000 rpm for 5 min at 4°C. The supernatants were removed and 250 μL of RIPA buffer (50 mM Tris HCL at pH 8.0, 150 mM NaCl, 0.5% Sodium Deoxycholate, 0.1% SDS, and fresh PMSF) were added to samples for incubation for 30 min with vortexing every 5 min. The tissue samples were then centrifuged at 13,000 rpm for 15 min at 4°C. The supernatants containing soluble proteins were transferred to a new tube. One hundred μL of RIPA was used to extract protein from pellet twice, and afterward combined with the supernatants. Protein concentrations were measured using Pierce BCA Protein Assay Kit (Thermo Scientific, Rockford, IL). Thirty μg of whole cell protein was electrophoresed on mini-protein TGX gels (Bio-Rad Laboratories, Hercules, CA) at a voltage of 90 V for 90 min. The protein bands were then transferred to a nitrocellulose membrane, where the transferred bands corresponding to MDM2 at 90 kDa, p53 at 53 kDa, p21 at 21 kDa, IGFBP-3 at 40/44 kDa, PTEN at 55 kDa, and β-actin at 43 kDa were respectively bound to monoclonal antibodies (Santa Cruz Biotechnology Inc., Santa Cruz, CA). Subsequently, the bound proteins were incubated with Thermal Scientific Pierce secondary antibody (Rockford, IL). Protein bands were visualized and quantified using FluorChem^™^ 8900 Advanced Imaging System (Alpha Innotech, San Leandro, CA). After the band density was standardized to the loading control of β-actin, the relevant band densities were divided by the corresponding acetone-treated sedentary control sample and reported as a percentage of the control.

### Statistical analysis

Student t-test was performed to analyze the significance of protein expression between sedentary control and exercise group with acetone vehicle control or TPA treatment. Two-way analysis of variance (ANOVA) was performed to assess the interaction between exercise and TPA stimulation. Minimum significance level was set at 0.05.

## Results

### Effects of exercise on MDM2 and p53 expression

**Fig 1:**
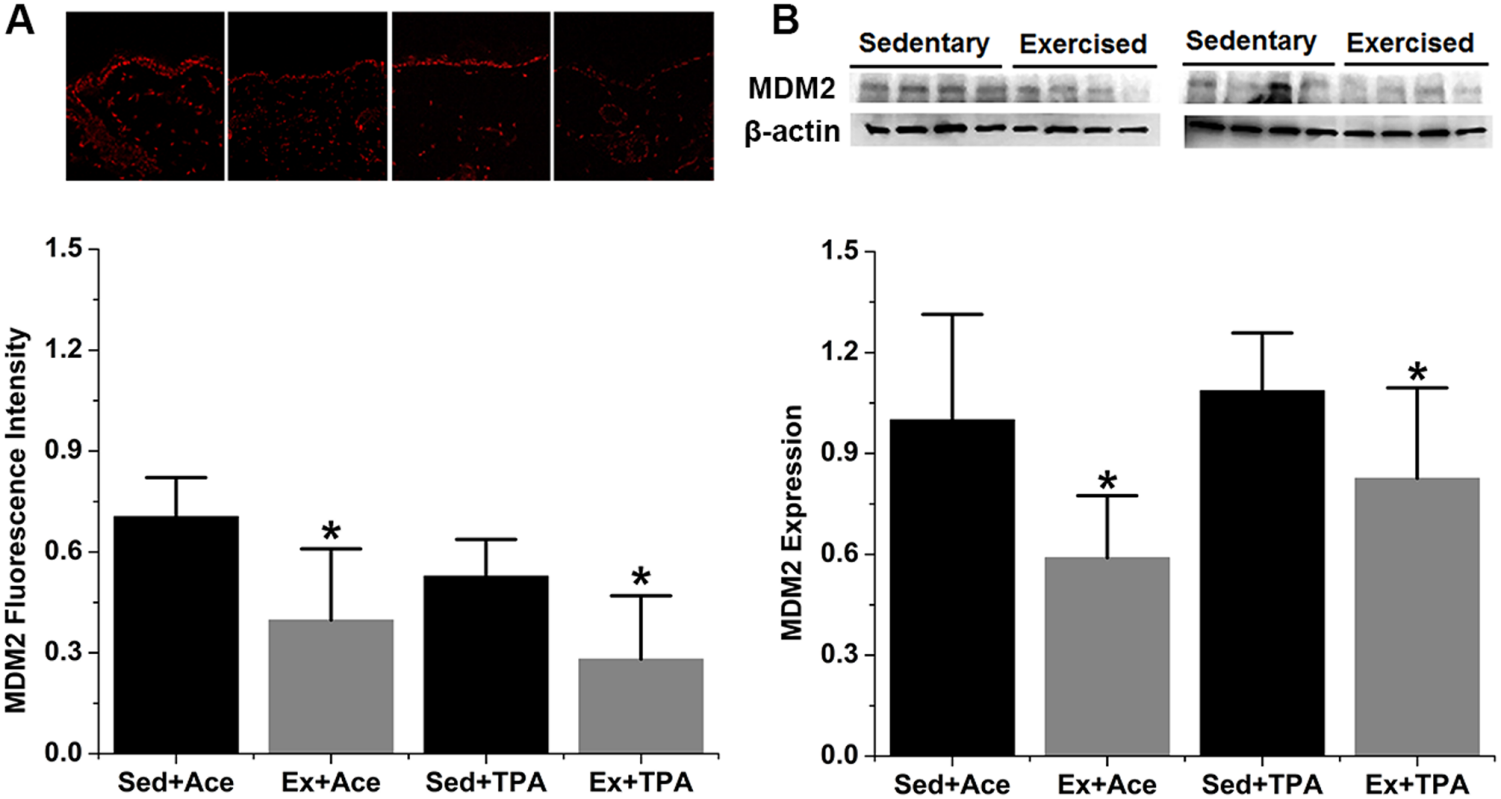
Exercise reduces MDM2 expression in both control and TPA-promoted skin epidermis of mice. SENCAR mice were performed treadmill exercise at 20 m/min, 60 min/day and 5 days/week for 10 weeks. The dorsal skin of each mouse was then treated with vehicle control or TPA at 3.2 nmol and analyzed for protein expression two hours after TPA treatment. (A) Representative confocal microscopy images of MDM2 fluorescence intensity (top) and quantification of IHC images (bottom). (B) Representative Western blots of MDM2 protein (top) and quantification of Western blot by densitometry (bottom). The data were normalized to the level of expression in one of the control and present as mean ± SD of three to four independent experiments. *, *p* < 0.05 versus sedentary control with vehicle or TPA treatment, respectively. Sed, sedentary control; Ex, exercised; Ace, acetone.

Representative images of fluorescence intensity of protein MDM2 in mouse dorsal tissues are shown in Fig 1A. The red stain corresponds to proteins to interest. Protein levels were obtained by analyzing the intensity of staining with images. When SENCAR mice were treated with acetone vehicle, MDM2 fluorescence intensity in exercised mice significantly decreased by 44% when compared with the sedentary control. Similarly, exercised mice with TPA treatment showed a decrease of 47% (*p* < 0.05) when compared to the counterpart control. This significant decrease of MDM2 determined by IHC was confirmed by Western blot results (Fig 1B). A significant decrease of MDM2 expression by 41% and 34% (*p* < 0.05) was observed within exercise group treated with acetone and TPA, respectively.

**Fig 2:**
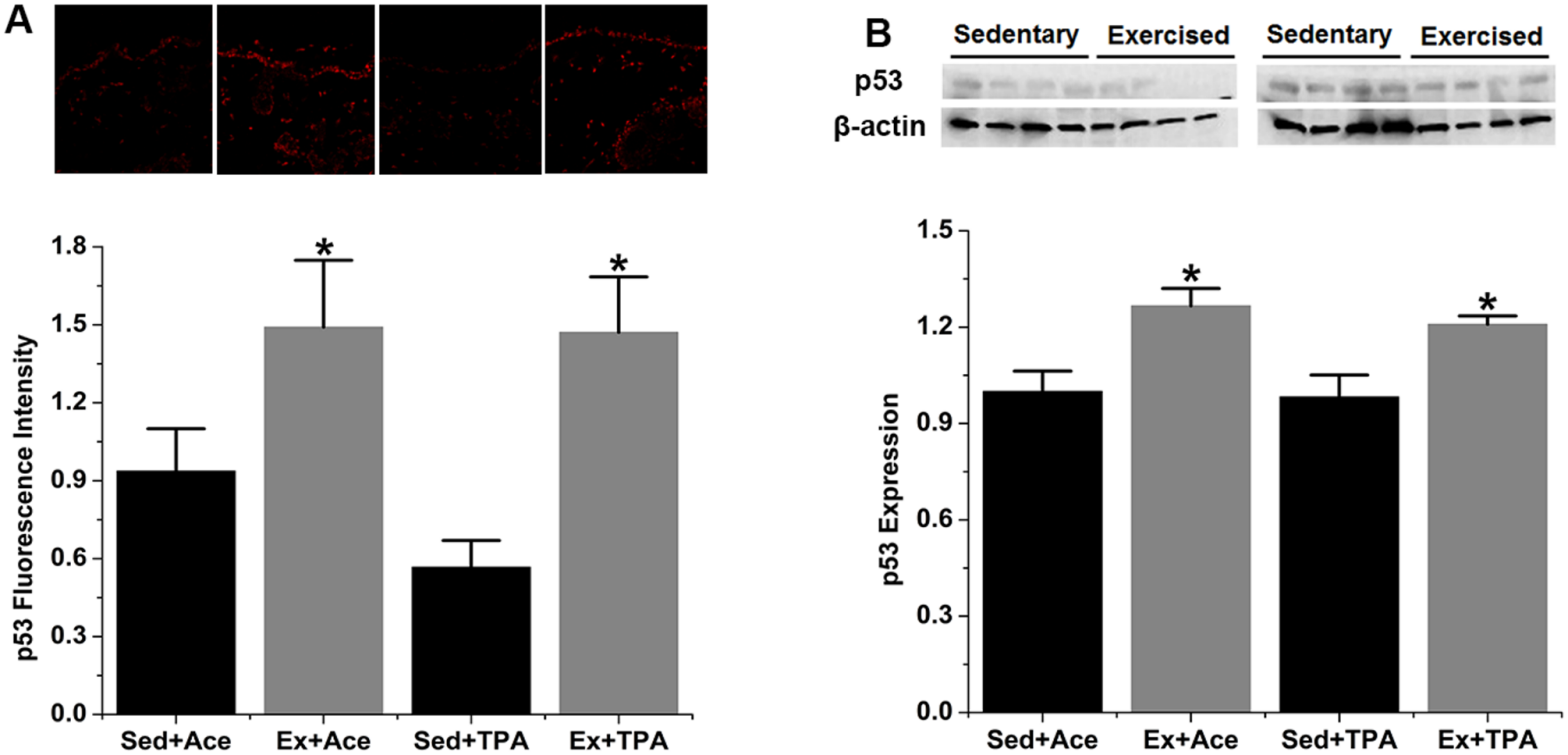
Exercise activates p53 expression in both control and TPA-promoted skin epidermis of mice. SENCAR mice were performed treadmill exercise at 20 m/min, 60 min/day and 5 days/week for 10 weeks. The dorsal skin of each mouse was then treated with vehicle control or TPA at 3.2 nmol and analyzed for protein expression two hours after TPA treatment. (A) Representative confocal microscopy images of p53 fluorescence intensity (top) and quantification of IHC images (bottom). (B) Representative Western blots of p53 protein (top) and quantification of Western blot by densitometry (bottom). The data were normalized to the level of expression in one of the control and present as mean ± SD of three to four independent experiments. *, *p* < 0.05 versus sedentary control with vehicle or TPA treatment, respectively. Sed, sedentary control; Ex, exercised; Ace, acetone.

Opposite to the decrease of MDM2, the fluorescence intensity of p53 protein prominently increased by 59% and 159% (*p* < 0.05) in exercised group treated with acetone and TPA, respectively (Fig 2A). Furthermore, Western blot showed a similar increase of p53 expression in exercise group under either acetone or TPA stimulation (Fig 2B).

### Effects of exercise on the expression of p53-transcripted proteins

**Fig 3:**
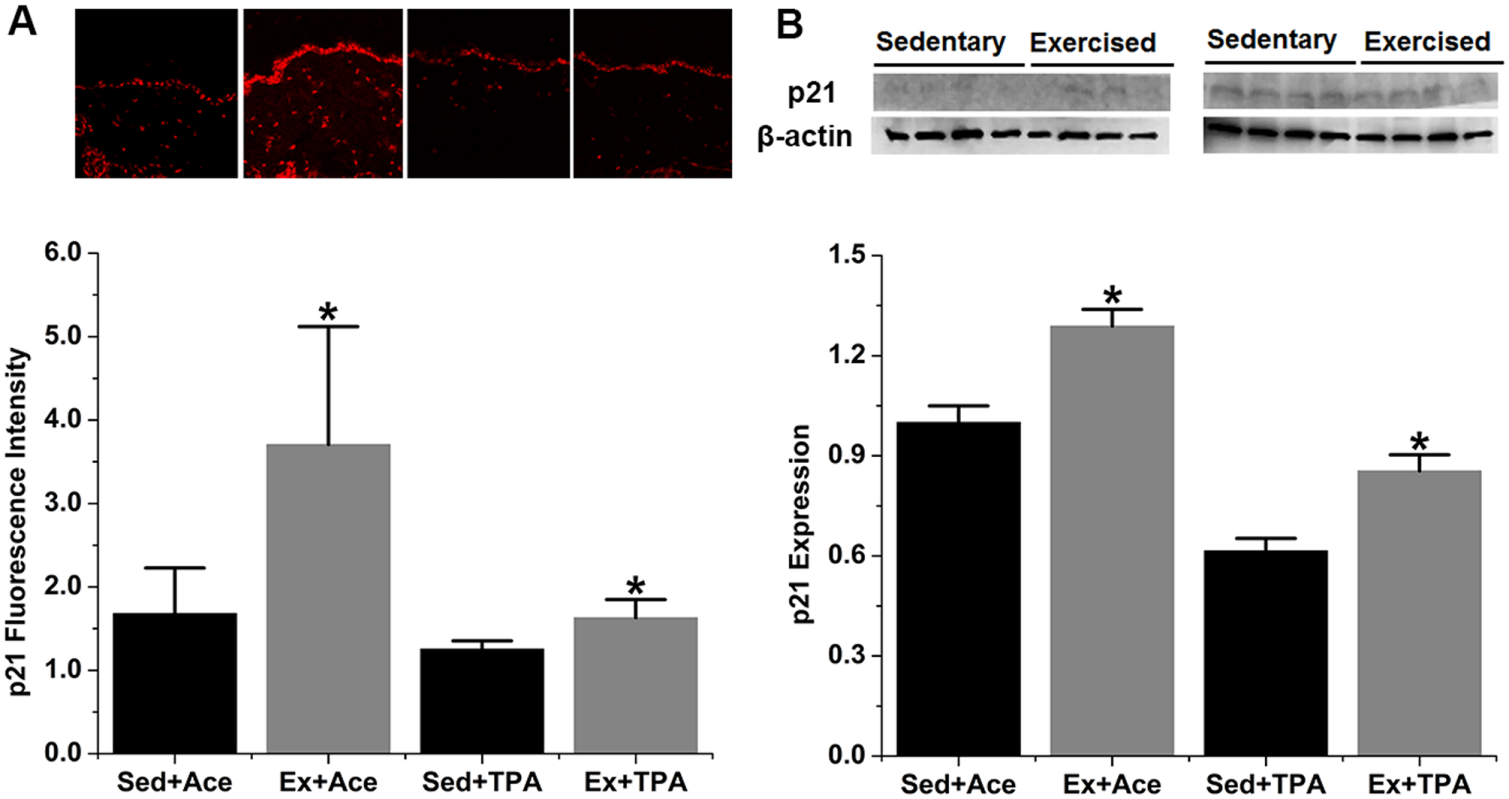
Exercise induces p21 expression in both control and TPA-promoted skin epidermis of mice. SENCAR mice were performed treadmill exercise at 20 m/min, 60 min/day and 5 days/week for 10 weeks. The dorsal skin of each mouse was then treated with vehicle control or TPA at 3.2 nmol and analyzed for protein expression two hours after TPA treatment. (A) Representative confocal microscopy images of p21 fluorescence intensity (top) and quantification of IHC images (bottom). (B) Representative Western blots of p21 protein (top) and quantification of Western blot by densitometry (bottom). The data were normalized to the level of expression in one of the control and present as mean ± SD of three to four independent experiments. *, *p* < 0.05 versus sedentary control with vehicle or TPA treatment, respectively. Sed, sedentary control; Ex, exercised; Ace, acetone.

**Fig 4:**
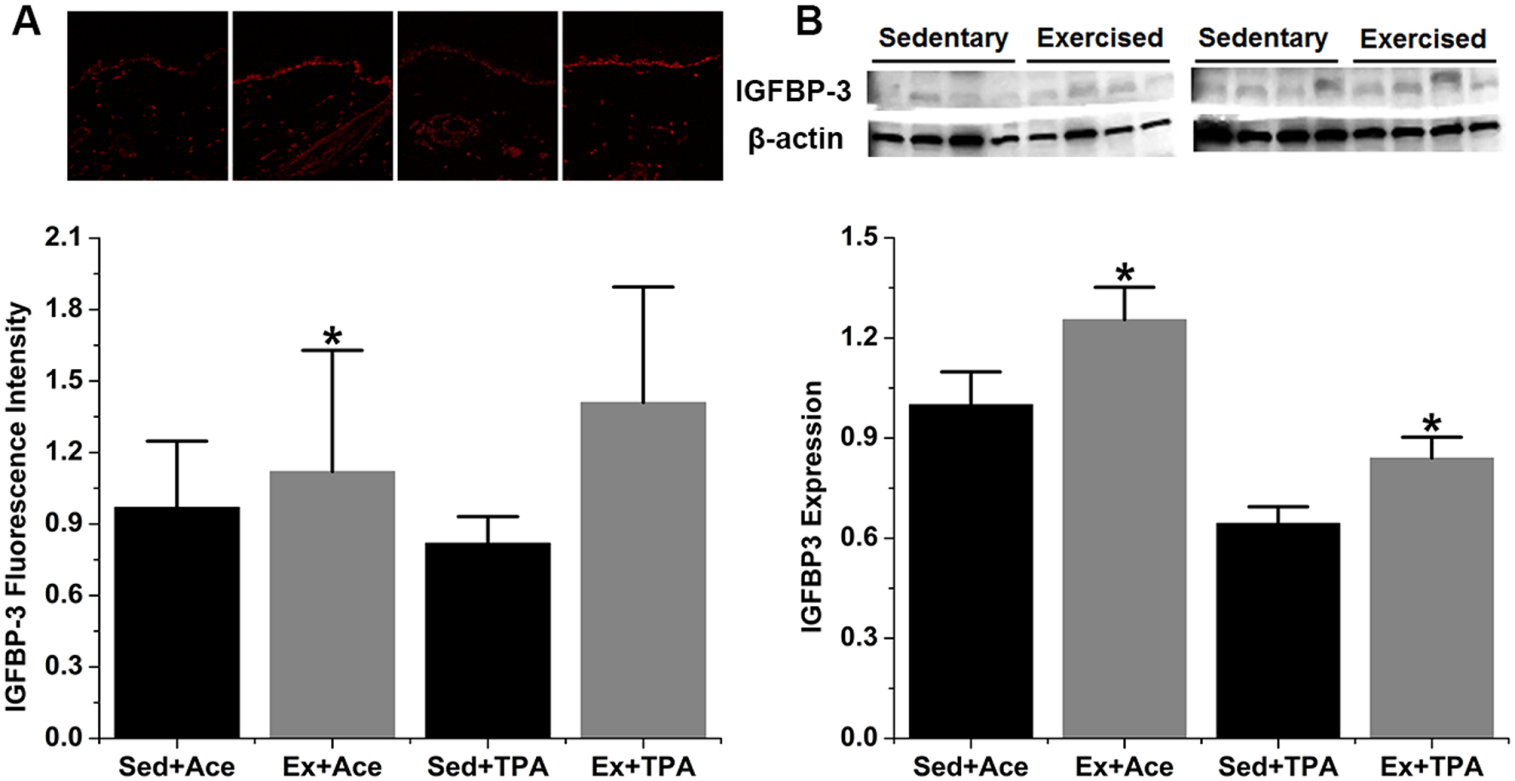
Exercise induces IGFBP-3 expression in both control and TPA-promoted skin epidermis of mice. SENCAR mice were performed treadmill exercise at 20 m/min, 60 min/day and 5 days/week for 10 weeks. The dorsal skin of each mouse was then treated with vehicle control or TPA at 3.2 nmol and analyzed for protein expression two hours after TPA treatment. (A) Representative confocal microscopy images of IGFBP-3 fluorescence intensity (top) and quantification of IHC images (bottom). (B) Representative Western blots of IGFBP-3 protein (top) and quantification of Western blot by densitometry (bottom). The data were normalized to the level of expression in one of the control and present as mean ± SD of three to four independent experiments. *, *p* < 0.05 versus sedentary control with vehicle or TPA treatment, respectively. Sed, sedentary control; Ex, exercised; Ace, acetone.

**Fig 5:**
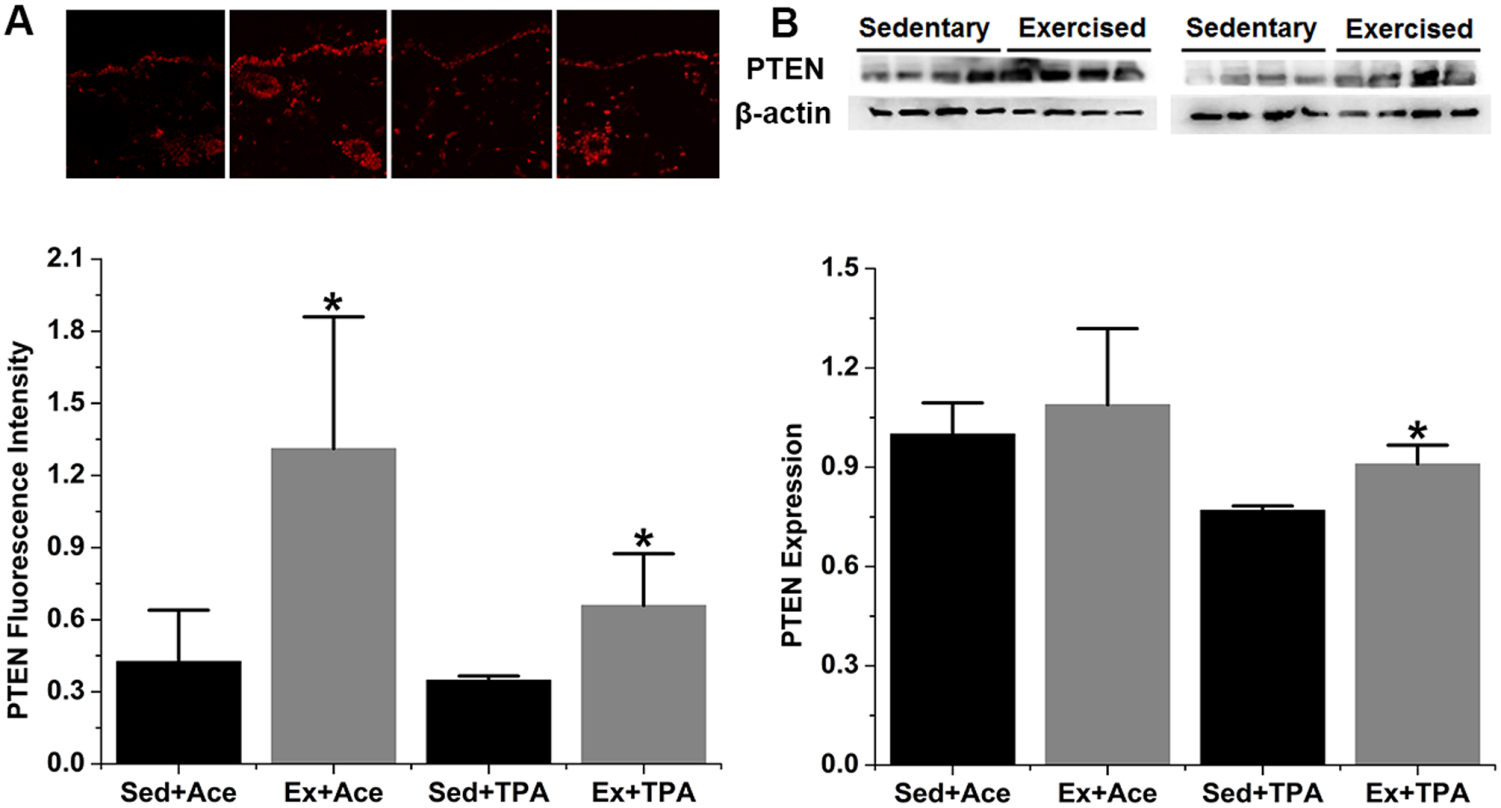
Exercise induces PTEN expression in both control and TPA-promoted skin epidermis of mice. SENCAR mice were performed treadmill exercise at 20 m/min, 60 min/day and 5 days/week for 10 weeks. The dorsal skin of each mouse was then treated with vehicle control or TPA at 3.2 nmol and analyzed for protein expression two hours after TPA treatment. (A) Representative confocal microscopy images of PTEN fluorescence intensity (top) and quantification of IHC images (bottom). (B) Representative Western blots of PTEN protein (top) and quantification of Western blot by densitometry (bottom). The data were normalized to the level of expression in one of the control and present as mean ± SD of three to four independent experiments. *, *p* < 0.05 versus sedentary control with vehicle or TPA treatment, respectively. Sed, sedentary control; Ex, exercised; Ace, acetone.

The expression of p53 downstream proteins, i.e., p21, IGFBP-3, and PTEN, were assessed by both IHC and Western blot. Elevated level of all the three proteins was observed in exercise group when compared with the sedentary control. As shown in Fig 3, p53-transcriptioned protein p21 was statistically enhanced by 121% (*p* < 0.05) as shown in IHC assay and 29% (*p* < 0.05) in Western blot in exercised animals with acetone treatment. Meanwhile, p21 increased in exercised group by 30% (*p* < 0.05) in IHC assay and 39% (*p* < 0.05) in Western blot under TPA promotion. IGFBP-3 expression is presented in Fig 4. A significant increase of IGFBP-3 by15% (*p* < 0.05) via IHC assay and 25% (*p* < 0.05) via Western blot was observed among acetone treated but exercised mice. During TPA stimulation, the increase of IGFBP-3 in exercised mice as measured by IHC was not significant, but it was significant by Western blot results. In terms of PTEN protein levels, a significant increase of 207% and 89% (*p* < 0.05) by acetone and TPA promotion, respectively, was found in IHC results (Fig 5A). Western blot was shown a significant increase of PTEN expression under TPA but not acetone treatment (Fig 5B).

### Interactive effects between exercise and TPA stimulation

The exercise and TPA interactive effects on all the five targeted proteins, i.e., MDM2, p53, p21, IGFBP-3, and PTEN, were further statistically calculated. An interaction between exercise and TPA on the decreased MDM2 or the increased p53 was found (*p* < 0.05), but the interactive effects on the p53-transcripted protein p21, IGFBP-3 and PTEN was not significant.

## Discussion

Our previous studies conducted in a mouse skin cancer model demonstrated a down-regulation of IGF-1 and IGF-1 signaling pathways was an essential cancer preventive target by exercise [9–14, 29]. To further understand the underlying mechanism, this study was focused on the effect of treadmill exercise on IGF-1 signaling regulators. Treadmill exercise at 20 m/min for 60 min/d, 5 d/week for 10 weeks was employed, which was recognized as a moderate physical activity based upon the maximal oxygen uptake [9–12] and had been shown greater protection against cancer risk [30]. As energy expenditure from exercise could be compensated by more calorie intake, exercised mice in this study were fed iso-caloric intake of the sedentary control. Therefore, the body weight of the exercised mice was significantly lower than the sedentary control (see S1 Dataset). Furthermore, a well-established mouse skin cancer model by using TPA as a cancer promoter was utilized. The TPA promotion period of 2 hour was selected because our previous time-course study onAP-1:DNA binding and c-Jun mRNA received optimal inducement after 2-hr TPA treatment [31]. It has also been confirmed in our previous studies that the 2-hr TPA promotion significantly induced the expression of TPA-promoted genes in skin adjusted by the basal levels in acetone treatment only [11].

To further illustrate how the IGF-1 and IGF-1 signaling was down-regulated by exercise, this study assessed p53 and p53-related IGF-1 pathway regulators. As expected, a significant decrease of MDM2 protein and increase of p53 protein was observed in exercised mice when compared with the sedentary control. It should be noted that exercise was reported to increase MDM2 levels in human and rodent muscles [32–33]. Although it is not clear of the conflict finding, it appears a diverse impact of exercise on MDM2 levels in different tissues. The exercise-induced p53 enhancement increased by 159% of the sedentary control after TPA promotion appears to be in agreement with a recent report by Higgins et al. that a dramatic increase of p53 protein was observed in the mouse lung tissues for lung tumor regression after a four-week wheel running exercise [34]. The reduction of MDM2 may be associated with the increase of p53, suggesting the suppression of p53 by MDM2 that may have been eliminated by exercise. However, there is no direct evidence in this study to support a causal linage between decreased MDM2 and increased p53 levels.

The activation of p53 following exercise was further confirmed by an increase of p53-dependent expression of p21, a well-known cyclin-dependent kinase inhibitor. Increased p53-p21 axis may induce cell cycle arrest and contribute, at least in part, to the cancer preventive mechanisms. Furthermore, a significant increase of both p53-transcripted IGFBP-3 and PTEN was found after treadmill exercise. IGFBP-3 is the most abundant IGF-1 binding protein that accounts for approximately 90% of bound IGF-1 in serum [35]. Increased expression of IGFBP-3 by exercise observed in this current study might be associated with less bioavailable IGF-1 as found in our previous studies [9, 11–12]. The p53-required induction of IGFBP-3 expression has been supported by a study using p53 knockout mice [36]. In addition to IGFBP-3, the increased expression of phosphoprotein PTEN, a well-known inhibitor of IGF-1-dependent PI3K-Akt pathway [37], might be directly related to IGF-1 pathway suppression. The p53-induced direct transcription of PTEN expression has also been reported previously [25]. As such, exercise-induced activation of p53 might reduce bioavailable IGF-1 via overexpressed IGFBP-3 and negatively regulate IGF-1 pathway through increased PTEN. A strength of this study was the quantification of each targeted protein by the same primary antibody for both IHC and Western blot techniques. A similar trend of change in protein detection was generally demonstrated by both methods. While there was a consistent change of MDM2, p53, and p21 between both methods, however, the significance of IGFBP-3 and PTEN was inconsistent between IHC and Western blot. IHC did not show a significant increase of IGFBP-3 in TPA-promoted condition, but it provided a basic characterization of the distribution of each targeted protein in addition to quantitative measurement. The basal level of PTEN expression, on the other hand, did not significantly detected by Westerns blot. Although Western blot is generally more quantitative and sensitive than IHC, it should be noted the skin epidermis might be diluted by other types of cells and thus cause some variation. Another strength of this study was compared the effects of exercise on both basal level and TPA-promoted expression of the targeted proteins. Considering the potential roles of these p53-related tumor suppression proteins, it should be interesting to know the impact not only on the situation of a cancer risk induced by TPA but also on the basal level of a normal environment for prevention. An interaction between exercise and TPA on the decreased MDM2 and increased p53 observed in this study indicates a possible synergy or joint effects, suggesting a discernibly larger effect of exercise during high cancer risk than that in a normal or low cancer risk.

**Fig 6:**
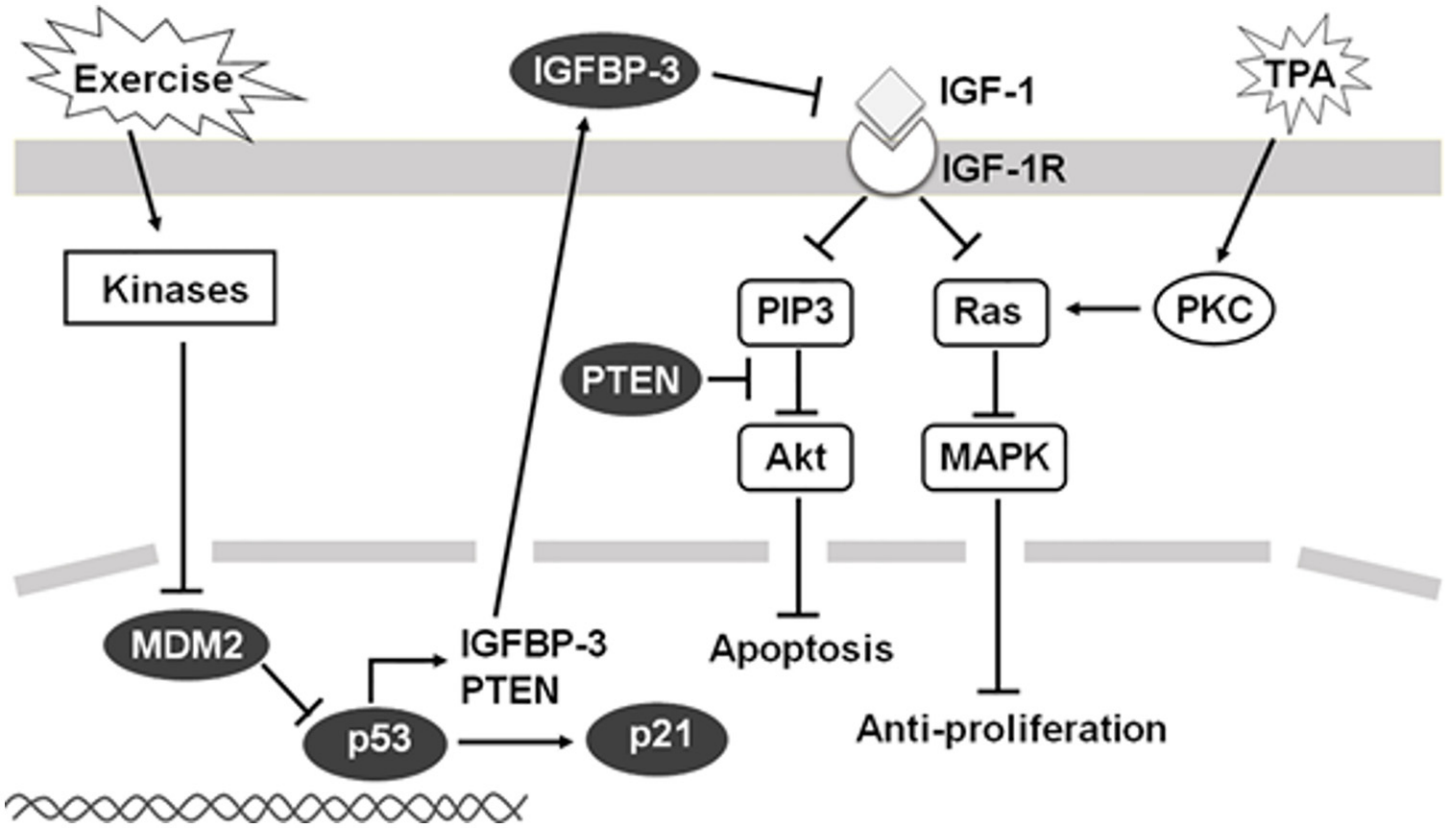
Overview of the p53 and p53-related IGF-1 signaling pathway regulators through which exercise may impact for cancer prevention.

Taken together, this current study demonstrated the effects of exercise on p53-related IGF-1 signaling regulators with respect to potential cancer preventive mechanisms. As concluded in Fig 6, exercise may activate p53 by reduced MDM2 expression, subsequently resulting in overexpression of p21, IGFBP-3, and PTEN. Both IGFBP-3 and PTEN may further reduce bioavailable IGF-1 and IGF-1-dependent pathway. A negative regulation of IGF-1 pathway through p53 and p53-related regulators may thus inhibit IGF-1 pathways and thus contribute to the observed exercise-induced cancer prevention in this mouse skin cancer model.

## Supporting Information

S1 Dataset Body weight change. (XLSX)

## Abbreviations

IGF-1: insulin-like growth factor-1
TPA: 12-0-tetradecanoylphorbol-13-acetate
MDM2: mouse double minute 2 homolog
IGFBP-3: insulin-like growth factor-binding protein 3
PTEN: phosphatase and tensin homolog
IHC: immunohistochemistry
PMSF: phenylmethanesulfonyl fluoride
RIPA: radioimmunoprecipitation assay
SDS: sodium dodecyl sulfate
BCA: bicinchoninic acid assay
ICE: interleukin-1β-converting enzyme
PI3K: phosphatidylinositide 3-kinases
Akt: protein kinase B
PIP2: phosphatidylinositol 4,5-bisphosphate
AP-1: activator protein 1
